# Bile acids in the lower airways is associated with airway microbiota changes in chronic obstructive pulmonary disease: an observational study

**DOI:** 10.1136/bmjresp-2024-002552

**Published:** 2024-12-18

**Authors:** Jose A Caparros-Martin, Montserrat Saladié, S Patricia Agudelo-Romero, Kristy S Nichol, F Jerry Reen, Yuben P Moodley, Siobhain Mulrennan, Stephen Stick, Peter A B Wark, Fergal O’Gara

**Affiliations:** 1 Wal-yan Respiratory Research Centre, The Kids Research Institute Australia, Nedlands, Western Australia, Australia; 2 Curtin Health Innovation Research Institute (CHIRI), Curtin University, Bentley, Western Australia, Australia; 3 The University of Western Australia, Perth, Western Australia, Australia; 4 European Virus Bioinformatics Centre, Jena, TH, Germany; 5 Immune Health Program, Hunter Medical Research Institute, University of Newcastle, Newcastle, New South Wales, Australia; 6 School of Microbiology, University College Cork, Cork, Ireland; 7 Synthesis and Solid-State Pharmaceutical Centre, University College Cork, Cork, Ireland; 8 Centre for Respiratory Health, School of Biomedical Science, The University of Western Australia, Nedlands, Western Australia, Australia; 9 Cell Biology Group, Institute for Respiratory Health, Nedlands, Western Australia, Australia; 10 Department of Respiratory Medicine, Fiona Stanley Hospital, Murdoch, Western Australia, Australia; 11 Institute of Respiratory Health and Medical School, The University of Western Australia, Nedlands, Western Australia, Australia; 12 Department of Respiratory Medicine, Princess Margaret Hospital for Children, Perth, Western Australia, Australia; 13 Faculty of Medicine Nursing and Health Sciences, Monash University, Prahran, Victoria, Australia; 14 BIOMERIT Research Centre, School of Microbiology, University College Cork, Cork, Ireland

**Keywords:** Pulmonary Disease, Chronic Obstructive, COPD Pathology

## Abstract

**Background:**

Chronic obstructive pulmonary disease (COPD) is a complex disorder with a high degree of interindividual variability. Gastrointestinal dysfunction is common in patients with COPD and has been proposed to influence the clinical progression of the disease. Using the presence of bile acid(s) (BA) in bronchoalveolar lavage (BAL) fluid as a marker of gastric aspiration, we evaluated the relationships between BAs, clinical outcomes and bacterial lung colonisation.

**Methods:**

We used BAL specimens from a cohort of patients with COPD and healthy controls. BAs were profiled and quantified in BAL supernatants using mass spectrometry. Microbial DNA was extracted from BAL pellets and quantified using quantitative PCR. We profiled the BAL microbiota using an amplicon sequencing approach targeting the V3-V4 region of the 16S rRNA gene.

**Results:**

Detection of BAs in BAL was more likely at the earliest clinical stages of COPD and was independent of the degree of airway obstruction. BAL specimens with BAs demonstrated higher bacterial biomass and lower diversity. Likewise, the odds of recovering bacterial cultures from BAL were higher if BAs were also detected. Detection of BAs in BAL was not associated with either inflammatory markers or clinical outcomes. We also observed different bacterial community types in BAL, which were associated with different clinical groups, levels of inflammatory markers and the degree of airway obstruction.

**Conclusion:**

Detection of BAs in BAL was associated with alterations in the airway bacterial communities. Further studies are needed to evaluate whether BAs in BAL can be used to stratify patients and predict disease progression trajectories.

**WHAT IS ALREADY KNOWN ON THIS TOPIC**

- Gastrointestinal manifestations are associated with clinical heterogeneity and disease progression in chronic obstructive pulmonary disease (COPD).
- The lung microbiota originates from the aspiration of bacteria residing in the upper airways, and its composition represents a source of clinical heterogeneity in COPD.

**WHAT THIS STUDY ADDS**

- Bile acid detection in bronchoalveolar lavage from patients with COPD was associated with higher bacterial biomass and lower diversity but not with clinical outcomes.
- Different microbial community types were associated with contrasting clinical outcomes in COPD.

**HOW THIS STUDY MIGHT AFFECT RESEARCH, PRACTICE OR POLICY**

- This study offers valuable insights into the established connections between gastrointestinal dysfunction and the progression of COPD, suggesting that aspiration of gastric contents may impact the microbial ecology in the COPD airways.

## Introduction

Chronic obstructive pulmonary disease (COPD) is a heterogenous lung condition characterised by persistent respiratory symptoms resulting from ongoing physiological alterations and structural changes in the airways and/or alveoli, leading in some cases to progressive airflow limitation.^1^ The aetiology of COPD is heterogenous and has been traditionally linked to long-term exposure to tobacco smoke and airborne pollutants that irritate the airways.^1^ However, factors such as abnormal lung development, a history of uncontrolled asthma, early life respiratory infections and other pathobiological mechanisms, such as the altered regenerative potential of alveolar progenitor cells, have been linked to an increased risk of developing COPD.^1 2^ The clinical evolution of COPD is dictated by worsening episodes of variable aetiology and severity called exacerbations.^3^ Other factors, such as microbial dynamics and airway colonisation, airway hyper-reactivity and immune and gastro-oesophageal dysfunction have all been shown to influence disease severity and progression.^3^

The prevalence of functional gastrointestinal pathology in patients with chronic lung disease is high.^4^ A common extrapulmonary manifestation in patients with COPD is gastro-oesophageal reflux disease (GERD).^5^ Numerous epidemiological studies have shown a robust relationship between GERD and increased incidence of COPD exacerbations.^6^,^9^ This suggests that reflux may modulate disease severity in COPD. The mechanisms through which GERD could aggravate the pathophysiology of COPD are unknown, but it may involve the translocation of gastric metabolites and/or micro-organisms into the lower airways through a reflux-microaspiration process.^10^

Bile acids (BAs) are host metabolites involved in fat digestion. Apart from their role as fat emulsifiers, BAs have antimicrobial, endocrine and immune regulatory properties and modulate the expression of pathogenesis-related genes in bacterial pathogens.^11 12^ Several studies have identified BAs in bronchoalveolar lavage (BAL) fluid obtained from patients with different respiratory pathologies.^4^ When detected in BAL, BAs have been associated with pathophysiological events in chronic lung disease. Thus, the presence of BAs in BAL has been linked to airway inflammation in patients with cystic fibrosis,^13^,^16^ lung transplant (LTx) recipients^17^,^19^ and children with respiratory symptoms.^17^ In LTx recipients, detection of BAs in BAL is also a risk factor for allograft dysfunction and mortality.^18^

The lung microbiota originates from the aspiration of pharyngeal commensals,^20^ through a process that also calibrates the lung immune tone.^21^ In COPD, the composition of the lung microbiota represents a source of clinical heterogeneity. Different microbial assemblages or endotypes are linked to specific COPD clinical phenotypes, which determine treatment response and disease progression trajectories.^22 23^ Whether and how bacterial aspiration is clinically relevant for the progression of COPD is unknown.

In this study, we evaluated whether the detection of BAs in BAL is associated with alterations in the BAL-associated bacterial and inflammatory profiles, as well as with clinical outcomes. We also evaluated the relationships between the bacterial communities present in BAL, inflammatory markers and clinical outcomes. We used a cohort of BAL specimens collected from clinically stable patients with COPD at different stages of the disease, and healthy controls with spirometry results in the normal range. The detection of BAs in BAL was more prevalent in patients with COPD during the early clinical stages of the disease and was independent of the severity of airway obstruction. In patients with COPD, detection of BAs in BAL was associated with alterations in the bacterial component of the BAL-associated microbiota, but not with clinical outcomes. Overall, our data suggest that the detection of BAs in BAL indicates processes associated with airway microbial colonisation in COPD.

## Methods

### Patient recruitment and collection of bronchoalveolar lavage samples

Patients were recruited through the respiratory clinic at John Hunter Hospital (Newcastle, New South Wales, Australia). The BAL samples were collected in accordance with the protocol outlined in a previous publication of the group.^24^ In all cases, bronchoscopy was conducted based on clinical indications, and participants provided informed consent for the collection of samples on meeting the inclusion criteria. Patients with COPD were in stable condition, without change in medications or experiencing acute exacerbations in the preceding 6 weeks. Healthy controls had normal spirometry results (forced expiratory volume in 1 s (FEV1)>80% predicted, FEV1/forced vital capacity (FVC)>0.7) and had no history of chronic lung disease. Healthy controls were required to have refrained from smoking for the past 10 years and had a smoking history of less than 5 packages/year. None of the patients with COPD recruited for this study reported swallowing difficulties or were on continuous positive airway pressure therapy or other non-invasive ventilation for chronic respiratory failure. The number of biobanked BAL specimens with at least 2 mL of BAL fluid determined the sample size in this study.

### Isolation of microbial DNA from BALF, 16S metabarcoding, sequencing of the amplicon pools and processing of the sequencing data

We obtained cell pellets from 163 out of the 166 BAL fluid (BALF specimens through high-speed centrifugation of 2 mL of BAL. Reagent-related contaminants were controlled by sequencing negative extraction controls. Extraction of microbial DNA and amplicon-based bacterial profiling targeting the V3-V4 region of the 16S rRNA gene were performed blinded to clinical data as we described.^25^ In this project, we obtained 19 236 207 single reads with a good base calling accuracy (96% of sequences with mean Phred-like Q-score greater than or equal to 30). Raw sequencing data was processed as we described.^25^ Quality-based pre-processing resulted in 8 538 607 paired-end reads (average length 405 bp), with >94% of the reads having a mean Phred-like Q-score greater than or equal to 35. Pre-processed joint reads were analysed following the SILVAngs pipeline^26 27^ as we described.^25^ For taxonomic classification we used the last release of the standardised SILVA SSU taxonomy (release 138.1) as reported.^25^ Following this processing pipeline, amplicon sequencing data was assigned to 1280 operational taxonomic units.

### Removal of the potential background contaminants determined by negative extraction controls

Bacterial burden in negative processing controls was used to determine the background noise associated with our experimental conditions. For 17 DNA extracts, we did not have enough DNA for assessing bacterial load and the taxonomy profiles from these BAL samples were therefore removed from the final data set. We set the background cut-off to 4100 16S DNA copies µL^−1^ of DNA extract, which lies 3 SD from the mean bacterial load in the negative extraction controls (mean 1722.58, SD 777.86, 16S DNA copies µL^−1^ DNA extract) (online supplemental figure S1). In our data set, 104 DNA extracts from BAL had bacterial burden above the background cut-off. These 104 DNA extracts were therefore considered for further analysis on the basis that, the associated microbial profiles, are more likely to represent a true biological signal. The taxonomic table was then processed to eliminate singletons, reads classified as chloroplast (mean relative abundance (SD), 0.005% (0.03)), mitochondria (0.00004% (0.0002)) or eukaryota taxa (0.0000006% (0.000009)) and no classified reads (0.001% (0.002)). Taxa representing less than 0.01% across all samples were also removed. Potential contaminants introduced during the processing of the samples were estimated using the R package *decontam*^28^ as we previously described.^25^ We detected and removed from the OTU table eight OTUs potentially arising from environmental contamination (online supplemental table S1). These filtering steps removed 1127 OTUs from the unfiltered data set, resulting in an OTU table with 153 unique taxonomy paths. Procrustes analysis confirmed that these filtering steps did not significantly impact the overall structure of the unfiltered microbial profiles (online supplemental table S2 and figure S2). We obtained a good correspondence between clinical microbiology data from BAL cultures and the taxonomic profiles obtained using molecular methods (online supplemental table S3).

### Quantitative analysis of bacterial burden in BAL

We used a TaqMan probe to estimate bacteria burden in DNA extracts as described in our previous publication.^25^

### Quantitative profiling of bile acids in BAL

We extracted BAs from spun BAL supernatants using solid-phase separation.^13^ For identification and quantification of the different BA analytes we used mass spectrometry as we previously described.^13 29^

### Statistical analyses

Statistical analyses and graphical representations were done in R (V.4.0.2). The normality of data distribution was evaluated using the Shapiro-Wilk test. When data did not follow a normal distribution, we evaluated differences between group medians using the Wilcoxon rank-sum test (WRST). Linear models were computed in R using the *lm* function. The assumption of homoscedasticity of the residuals and the absence of highly influential data points were evaluated using regression diagnostics. Shannon Diversity Index was calculated with the *diversity* function of the R package vegan. For cluster analysis of the BAL-associated bacterial communities we used Dirichlet multinomial mixtures models.^30^ We use the listwise deletion method to deal with missing data on analysed variables. Online supplemental table S4 indicates for each clinical variable, the number of participants with missing data within each clinical group. When multiple comparisons were involved in the analysis, p values were corrected using the Bonferroni method. The probability threshold for statistical significance was set at 0.05.

## Results

### Study population

This is a retrospective study of 166 BAL specimens collected from 108 patients with COPD (global initiative for chronic obstructive lung disease, GOLD1-4) and healthy non-smokers (nSC, 38) and healthy smoker (20) controls. Patients demographics are summarised in table 1 and inclusion criteria described in the Methods section. Briefly, compared with the control groups, patients with COPD were older and had a higher degree of airflow impairment in agreement with their GOLD stage category. Prescription records were available for 101 (93.5%) patients with COPD (online supplemental table S5). Most patients with COPD (83.2%) were prescribed a bronchodilator, of which 12% were also on corticosteroids. Bronchodilator use, and specifically treatment with either short-actin b2-agonists, long-acting b2-agonists with or without corticoids or long-acting muscarinic antagonists, were associated with GOLD categories (online supplemental table S5). Prescription of non-steroid anti-inflammatory drugs, or blood thinner medications were also associated with the GOLD stage (online supplemental table S5). Other medications, such as corticosteroid use, or medications to treat acid reflux and heartburn symptomatology including antacids and proton pump inhibitors (PPIs), were not associated with specific GOLD stages (online supplemental table S5).

**Table 1:** Patient demographics. The number of patients with data for each of the indicated variables is also shown. We used a joint *F*-test for assessing differences in the degree of airway obstruction and patient age. A χ^2^ test of independence was used to evaluate differences in patient sex across the different clinical groups

	FEV1/FVC, mean (SD); number of patients	Age, mean (SD); number of patients	Sex (% males); number of patients
GOLD1	72.29 (7.55); 21	64.52 (11.52); 25	40; 25
GOLD2	57.77 (10.70); 32	72.06 (7.14); 31	53; 32
GOLD3	49.53 (11.96); 36	69.03 (10.74); 36	67; 36
GOLD4	34.71 (9.31); 14	61.93 (10.85); 15	60; 15
nSC	76.15 (7.64); 33	57.63 (17.12); 38	42; 38
SC	75.76 (13.61); 17	51.85 (18.22); 20	55; 20
Statistical test	*F*=53.91***	*F*=9.17**	χ^2^ 10.82, n.s.

**p<0.01, ***p<0.001.

FEV_1_forced expiratory volume in 1 sFVCforced vital capacityGOLDglobal initiative for chronic obstructive lung diseasen.s.not significantnSChealthy non-smokers SChealthy smoker

### Detection of bile acids in BAL is associated with early stages of COPD

We detected BAs in 67 (40.4%) BAL specimens (median concentration (IQR), 0.24 (0.13–0.47) µM) (online supplemental table S6). The concentration of BAs in BAL from patients with COPD was higher than in healthy donors (median (IQR) in controls, 0 (0–0) µM; median (IQR) in patients with COPD, 0.007 (0–0.09) µM, WRST, p=0.0006, r=0.27) (online supplemental figure S3). In our cohort, detection of BAs in BAL was associated with patient age but not with the degree of airway obstruction (online supplemental figure S4). In patients with COPD, medicine prescription including bronchodilators, anti-inflammatory drugs or medicines to treat symptomatic reflux including antacid and PPIs, was not associated with the detection of BAs in BAL (online supplemental figure S5).

**Figure 1:**
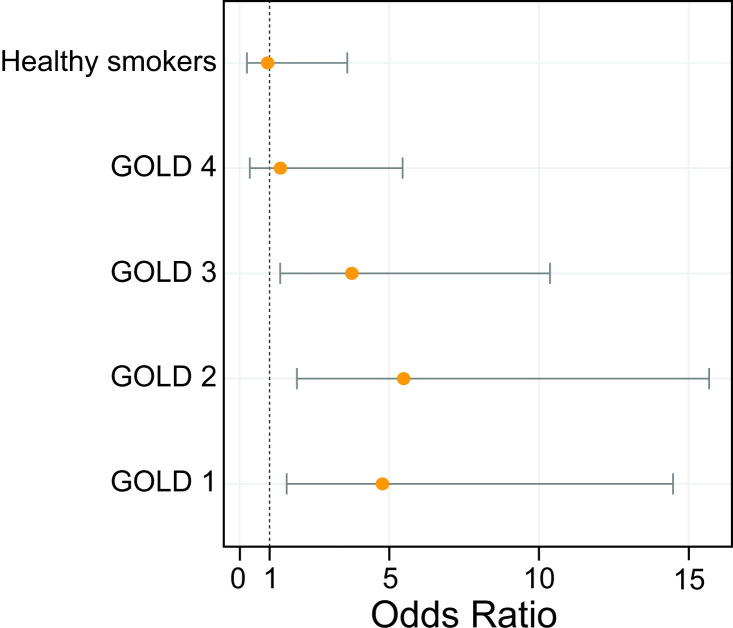
Forest plots showing the odds of detecting bile acids in bronchoalveolar lavage in the different population groups. OR are represented with a 95% pointwise CI. GOLD, global initiative for chronic obstructive lung disease.

Detection of BAs was more likely in BAL collected from patients with COPD than from healthy controls (OR (95% CI), 3.98 (1.90 to 8.33) p=0.0002). Eight of 38 non-smoker healthy controls (21%), 4 of 20 smoker healthy controls (20%), 14 of 25 patients with COPD from GOLD stage I (56%), 19 of 32 patients with COPD from GOLD stage II (59%), 18 of 36 patients with COPD from GOLD stage III (50%) and 4 of 15 patients with COPD from GOLD stage IV (27%), had BAs in BAL. Compared with the nSC group, the likelihood for detecting BAs in BAL was increased in early stages of COPD (OR (95% CI), GOLD1 4.77 (1.57 to 14.48) p=0.006, GOLD2 5.48 (1.91 to 15.69) p=0.001, GOLD3 3.75 (1.36 to 10.37) p=0.01, GOLD4 1.36 (0.34 to 5.45) p=0.66) (figure 1). We obtained similar results after controlling for the degree of airway obstruction (FEV_1_/FVC ratio) or patient age (online supplemental figure S6).

### Relationships between the presence of BAs in BAL and clinical outcomes

Patients with COPD with or without BAs in BAL, demonstrated similar levels of white cells in their lungs (median (×10^6^ cells/mL) (IQR), BAs detected 0.36 (0.25–0.57), no BAs detected 0.18 (0.16–0.63), WRST, p=0.3, r=0.24), with no differences in viability (median (×10^6^ cells/mL) (median (% of viable cells) (IQR)), BAs detected 90 (83–93), no BAs detected 92 (77–95), WRST, p=0.75, r=0.08). Similarly, there was no difference in the proportion of neutrophils in BAL (median (%) (IQR), BAs detected 68.4 (56.3–84.3), no BAs detected 62.6 (43.3–74.3), WRST, p=0.1, r=0.16) (online supplemental figure S7). Likewise, we did not observe a correlation between BAs concentrations and the percentage of neutrophils in BAL (online supplemental figure S8).

**Figure 2:**
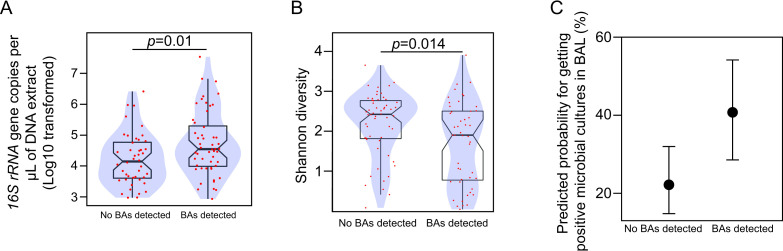
(**A**–**B**) Boxplots overlaid with density plots (blue) showing the relationship between BAs detection in BAL and bacterial load (**A**), and the diversity of the BAL-associated microbial communities (**B**). Individual values for each BAL specimen are represented with red dots. Notches in the boxplots represent a 95% CI for the sample median. Statistical significance was assessed using the Wilcoxon rank-sum test. For each comparison, the corresponding p value (p) is shown in the graph. (**C**) Marginal effect of BAs detection in BAL on the odds of observing positive microbial cultures from BAL with pointwise 95% CIs. Predicted probabilities were calculated from a logistic regression model. BAs, bile acid; BAL, bronchoalveolar lavage.

Conversely, detection of BAs was linked to a higher bacterial burden in BAL (median (log10 16S copies µL^−1^ of DNA extract) (IQR), BAs detected 4.54 (3.98–5.30), no BAs detected 4.14 (3.60–4.76), WRST, p=0.01, r=0.25) (figure 2A). In the case of the healthy control groups (smokers and non-smokers), the detection of BAs in BAL was not linked to differences in the proportion of infiltrated neutrophils, number and viability of white blood cells or bacterial load in BAL (online supplemental figure S7).

In patients with COPD, the presence of BAs in BAL was not correlated with clinical outcomes, including the ratio of FEV_1_ to FVC (median (IQR), BAs detected 57 (48–67), no BAs detected 52 (42–64), WRST, p=0.32, r=0.1), quality of life (COPD assessment test, CAT) (median (IQR), BAs detected 13 (10–16), no BAs detected 15 (7–19), WRST, p=0.95, r=0.01) and either number of exacerbations (median (IQR), BAs detected 4 (2–7), no BAs detected 3 (1–5), WRST, p=0.45, r=0.11), number of antibiotic rounds (median (IQR), BAs detected 2 (1–5), no BAs detected 2 (1–4), WRST, p=0.58, r=0.08), or number of oral corticosteroids rounds (median (IQR), BAs detected 1 (0–2), no BAs detected 0 (0–1), WRST, p=0.44, r=0.12) (online supplemental figure S9).

### Associations between the COPD BAL microbiota and clinical outcomes

We analysed the bacterial profiles of 104 BAL samples with bacterial biomass above the cut-off defined by the negative extraction controls (online supplemental figure S1). These taxonomic profiles were dominated by OTUs assigned to the *Streptococcus* (mean relative abundance (SD), 20.19% (17.79)) and *Haemophilus* (13.22% (28.58)) taxa (online supplemental figure S10).

In our cohort, alpha (within the sample) diversity was negatively associated with bacterial biomass (β −0.44, adjusted R^2^ 0.14, *F*(1,102)=17.20, p=0.00007) and the percentage of neutrophils in BAL (β −0.01, adjusted R^2^ 0.10, *F*(1,97)=12.41, p=0.0006) (online supplemental figure S11). We obtained similar results when the data from healthy controls (both smokers and non-smokers) was excluded from the linear models (online supplemental figure S12). Despite the observed relationships between microbial parameters and inflammation in BAL from patients with COPD, the degree of airway obstruction was not predicted by either the bacterial diversity (Shannon Index, β 2.66, adjusted R^2^ 0.02, *F*(1,70)=2.17, p=0.14), or the bacterial biomass in BAL (log10 16S copies µL^–1^ of DNA extract, β −1.70, adjusted R^2^ 0.00, *F*(1,70)=0.71, p=0.40) (online supplemental figure S13A,B). Likewise, quality of life or number of exacerbations were not correlated with either bacterial load or diversity in BAL (online supplemental figure S13C–F). Conversely, BAL specimens containing BAs demonstrated reduced diversity (median (IQR), BAs detected 1.90 (0.78–2.50), no BAs detected 2.42 (1.82–2.77); WRST, p=0.01, r=0.24) and increased odds for isolating micro-organisms in cultures from BAL (any micro-organism, OR 2.41 (1.15–5.02) p=0.02) (figure 2B,C). We did not observe a linear relationship between microbial diversity and the concentration of BAs in BAL (online supplemental figure S14).

**Figure 3:**
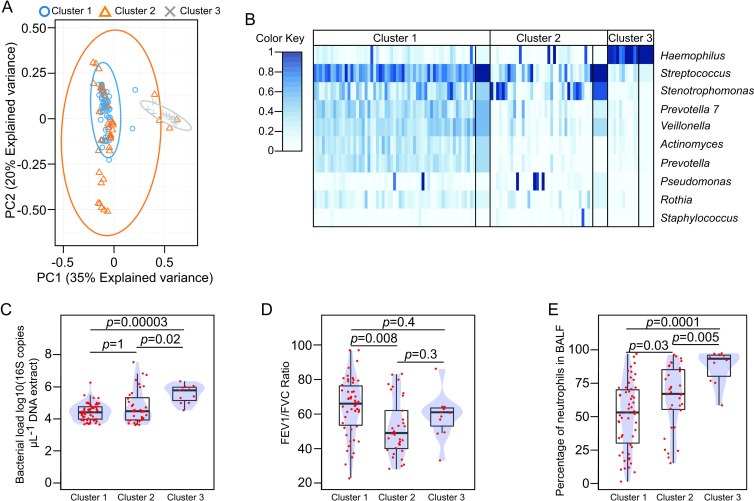
(**A**) Scatter plot showing the projection of the BAL-associated bacterial compositional profiles in the first two components of the principal component analysis. Samples are labelled accordingly with the cluster membership. (**B**) Heatmap summarising the top 10 OTUs ordered based on their contribution to the Dirichlet components. Narrow columns represent the square root of the OTU relative abundance, while wide columns represent the mean value of the Dirichlet component for each mixture. (**C–E**) Barplots overlaid with density curves representing differences in bacterial load (**C**), FEV1/FVC ratio (**D**), and the percentage of neutrophils in BAL (**E**), relative to the indicated microbial community cluster. Red dots represent observed values for each BAL specimen. Statistical significance was assessed using the Wilcoxon rank-sum test and p values corrected for multiple comparisons using the Bonferroni method. BAL, bronchoalveolar lavage; BALF, BAL fluid; FEV1, forced expiratory volume in 1 s; FVC, forced vital capacity; OTU, operational taxonomic units.

We used Dirichlet multinomial mixtures to classify the BAL-associated microbial communities and observed three different clusters or ecotypes (figure 3 and online supplemental figure S15). These three ecotypes showed contrasting levels of BAL-associated bacterial biomass (figure 3C). At the community level, two OTUs assigned to the *Streptococcus* and *Haemophilus* taxa were the more abundant entities defining each community-based cluster (online supplemental figure S16). Interestingly, each ecotype was associated with different clinical outcomes as well as specific clinical groups (figure 3D,E and online supplemental figure S17). Ecotypes I and II occurred in samples from both healthy controls and patients with COPD, although it is true that ecotype II was less likely in healthy controls (online supplemental figure S17). Both ecotype I and II were dominated by *Streptococcus*, however, ecotype II had a less evenly distributed community composition (figure 3B). Clinically, the transition from ecotype I to ecotype II was associated with a progressive increase in airway inflammation and a higher degree of obstructive impairment on spirometry (figure 3D,E). On the other hand, the *Haemophilus*-dominated community (ecotype III) was only observed in BAL specimens collected from patients with COPD (online supplemental figure S17). Ecotype III was also associated with a higher percentage of neutrophils in BAL (figure 3E). None of the three ecotypes was significantly associated with either specific GOLD stages or BA detection in BAL (online supplemental figure S17).

## Discussion

Gastrointestinal manifestations are commonly observed in patients with COPD, and they have been linked to disease heterogeneity.^6^ Thus, GERD, which could lead to the aspiration of gastric contents, is associated with a ‘frequent exacerbator’ phenotype.^6^,^9^ In patients with COPD, several of the physiological barriers preventing the gastric contents from reaching the airways are impaired.^4^ Moreover, most patients with COPD smoke or are frequently treated with beta-agonists and theophyllines, which affect the physiology of the oesophageal sphincters.^4^ These functional defects are therefore expected to facilitate reflux and aspiration of gastric contents, and they likely represent a functional link between the gastrointestinal tract and respiratory disease. In this study, we evaluated the clinical significance of the presence of BAs in BAL and their impact on BAL-associated microbiota. BAs are gastric metabolites that, when observed in the lower airways, are considered a gastric marker for aspiration.^10^ In our cohort, the likelihood of detecting BAs in BAL was significantly increased in patients with early-stage COPD disease compared with healthy individuals. This observation was independent of the degree of airway obstruction present but was positively linked to patient age. Past publications have reported contrasting results regarding a relationship between the presence of BAs in BAL, and lung function.^13 15 17 31^ This discrepancy between studies suggests that while lung deterioration could facilitate BAs detection in BAL (eg, mucociliary clearance impairment), extrapulmonary mechanisms are also involved.^4^ Similarly, we did not observe a relationship between the detection of BAs in BAL from patients with COPD and the prescription of medicines known to affect the physiology of the oesophageal sphincters,^4^ or medications to treat symptomatic reflux such as antacids and PPIs. A previous study reported similar results regarding PPI use.^17^ The authors also demonstrated that, rather than reflux burden, delayed gastric emptying was significantly associated with the presence of BAs in BAL,^17^ further highlighting the complexity of the potential mechanisms linking gastrointestinal manifestations with lung disease progression. We also detected BAs in 20–21% of BAL samples collected from healthy controls, and this was independent of smoking status. A previous study reported BAs in 13% of sputum samples from healthy individuals,^14^ supporting the notion that BAs reach the airways in the absence of a respiratory pathology. Likewise, this 10–20% detection range in healthy subjects could be explained by the estimated 10–30% prevalence of GERD in the Western population.^32^

Persistent airway inflammation is a common feature in chronic lung disease. Although tobacco smoke is the major driver of inflammation in COPD lungs, inflammatory markers remain elevated even after the cessation of smoking.^33^ Thus, additional factors are likely involved in maintaining the inflammatory milieu in COPD lungs. BAs have been robustly linked to inflammation and poor clinical outcomes in different respiratory disorders,^1013^,^15 17^ and they could provide a link between GERD and COPD disease severity.^6^ In our study, the percentage of neutrophils in COPD BAL regardless of BAs presence was comparable, suggesting that BAs are not involved in establishing the inflammatory landscape of COPD lungs. Similarly, we did not observe a relationship between the presence of BAs in BAL and clinical outcomes. These results are in line with a previous study whose findings did not support the role of BAs in COPD exacerbations.^34^ We however observed a robust association between BAs and ecological parameters in BAL. Aspiration can shape the COPD lung microbiota,^35^ and is a mechanism for pulmonary colonisation.^20^ Likewise, bacterial, and viral infections are known triggers of exacerbations in COPD.^36^ In our study, COPD BAL samples with BAs demonstrated higher bacterial load and lower diversity than specimens without BAs. Additionally, the probability of a positive microbial culture from BAL was significantly increased if BAs were also detected. Considering this, future studies are required to clarify whether the known relationship between GERD and COPD exacerbations could be explained by reflux increasing the risk of aspiration of microbial pathogens.

The lung microbiome plays an important role in the progression of COPD.^22 23^ We found that BAL bacterial biomass and diversity were negatively correlated with the percentage of infiltrated neutrophils. However, clinical outcomes were independent of bacterial community descriptors in BAL. This unexpected observation supports previous studies demonstrating that clinical responses in COPD depend on particular microbial community signatures,^22 23^ rather than on the overall structure of the bacterial community.

We also examined the microbial communities present in BAL. Using cluster analysis and likelihood estimators, we identified three community types, that were correlated with contrasting degrees of airway obstruction and inflammation and different clinical groups. We found that *Haemophilus*-dominated communities were characterised by elevated biomass, and were also linked to the highest percentage of neutrophils in BAL. This community type was exclusively observed in COPD BAL samples. This result is in line with previous work in COPD demonstrating that *Haemophilus* dominance is consistently associated with neutrophilic inflammation.^22 23^ We also identified two *Streptococcus*-dominated microbial community types, which could represent different states of the balanced microbiome profile previously described in COPD sputum.^22 23^ *Streptococcus* dominance was observed in BAL from both healthy controls and patients with COPD, and it was not linked to specific clinical stages of the disease. Interestingly, both ecotypes showed contrasting patterns of microbial distribution, with the less evenly distributed signature associated with a progressive increase in airway obstruction and inflammation. Detection of BAs in BAL did not correlate with specific microbial assemblages. This result suggests that although bacteria and BAs could access the airways through the same physiological process, additional factors determine the type of bacterial community thriving in the lungs.

An obvious limitation of our study is its observational nature, which does not allow causality to be established. Likewise, participants did not undergo gastro-oesophageal reflux monitoring. Thus, we cannot confirm the physiological alteration mediating BAs intrusion into the airways. Although the physiological process underlying the translocation of BAs into the lungs is not relevant to the associations shown in the present study, BAs in BAL are considered a marker of gastric aspiration.^10^ Lastly, BAs and the microbial profiles could be confounded by salivary contamination during bronchoscopy. Although we cannot rule out this possibility, our results demonstrating an association between the detection of BAs and specific clinical groups are unlikely to be driven by a contamination signal.

Overall, in this observational study, we show that the detection of BAs in BAL is associated with the early stages of COPD, and correlated with parameters of airway ecology, but not with clinical outcomes. Future longitudinal studies are needed to determine whether the presence of BAs in BAL at the early clinical stages of COPD is a useful approach to stratify patients with COPD, such as identifying those on a worse disease progression trajectory. Additionally, further research should explore whether the established links between GERD and COPD exacerbations are associated with unstable lung microbial communities and an increased susceptibility to acquiring pro-inflammatory microbial communities in the lungs.

## supplementary material

10.1136/bmjresp-2024-002552 online supplemental file 1

## Data Availability

Data are available upon reasonable request.
